# An Iterative Design Approach to Development of an Ex Situ Normothermic Multivisceral Perfusion Platform

**DOI:** 10.3390/jcm14134620

**Published:** 2025-06-30

**Authors:** L. Leonie van Leeuwen, Matthew L. Holzner, Ceilidh McKenney, Rachel Todd, Jamie K. Frost, Sneha Gudibendi, Leona Kim-Schluger, Thomas Schiano, Sander Florman, M. Zeeshan Akhtar

**Affiliations:** Organ Perfusion Unit, Recanati/Miller Transplantation Institute, Icahn School of Medicine at Mount Sinai, 1468 Madison Ave, New York City, NY 10029, USAsneha.gudibendi181@gmail.com (S.G.); zeeshan.akhtar@mountsinai.org (M.Z.A.)

**Keywords:** organ preservation, machine perfusion, multivisceral transplantation

## Abstract

**Background/Objectives:** Challenges in normothermic machine perfusion (NMP) remain, particularly concerning the duration for which individual organs can be safely preserved. We hypothesize that optimal preservation can be achieved by perfusing organs together in a multivisceral block. Therefore, our aim was to establish a platform for ex situ multivisceral organ perfusion. **Methods:** Multivisceral grafts containing the liver, kidneys, pancreas, spleen, and intestine were obtained from Yorkshire pigs. Three generation (gen) set-ups were tested during the iterative design process, and minor changes were made throughout. Gen 1 (*n* = 4) used a custom-designed single perfusion circuit. Gen 2 (*n* = 3) employed a dual perfusion circuit. Gen 3 (*n* = 4) featured a single perfusion circuit with an optimized basin and reservoir. Grafts underwent NMP using an autologous blood-based perfusate, while hemostatic parameters and function were assessed. **Results:** Comparing Gen 1 versus Gen 3, the mean aortic flow improved (1.018 vs. 2.089 L), resistance decreased (0.224 vs. 0.038), urine output increased (51.90 vs. 271.3 mL), oxygen consumption rose (43.56 vs. 49.52 mL O_2_/min), perfusate lactate levels dropped (10.44 vs. 3.10 mmol/L), and the pH became more physiological (7.27 vs. 7.30). Cellular injury trended lower in Gen 3. Histological evaluation demonstrated minimal differences in Gens 2 and 3. **Conclusions:** We demonstrate the feasibility of abdominal multivisceral NMP for up to 8 h. Adequate arterial flow, stable perfusate pH, and high oxygen consumption in setup 3 indicated organ viability. Multivisceral perfusion may serve as a plat-form for long-term NMP.

## 1. Introduction

Medical technologies continue to evolve in response to the growing complexity of patient care. In the field of transplantation, normothermic machine perfusion (NMP) has emerged as a transformative approach, enabling ex situ preservation, assessment, optimization, and repair of various organs, including the heart, lungs, liver, and kidneys [1,2,3,4,5,6]. Whether applied independently or combined with technologies like normothermic regional perfusion (NRP) and hypothermic machine perfusion (HMP), NMP is actively expanding the pool of viable organs for transplant.

Despite these advancements, challenges remain, particularly in extending safe preservation duration. Prolonged liver perfusion (beyond 24–36 h) often requires dialysis filters to clear waste products [7]. Additionally, preserving the kidneys, pancreas, and intestines under normothermic conditions remains complex. In the case of kidneys, the rationale for choosing normothermic over hypothermic perfusion is still unclear, and the organ’s response to metabolic demands during NMP differs significantly from both hypothermic and in situ conditions as well as per device and protocol used [8,9]. The pancreas, being a particularly susceptible low-flow organ, is prone to substantial edema and injury during normothermic perfusion, making it difficult to maintain without damage [10]. Similarly, normothermic bowel perfusion is rarely achieved beyond six hours [11,12], making static cold storage or HMP more reliable for certain organs. However, normothermic environments offer the most suitable conditions for detailed biological assessments and the implementation of therapies, including cellular and regenerative treatments.

We hypothesize that *en bloc* organ preservation could overcome the limitations of isolated perfusion by better replicating physiological in situ conditions, enabling viable long-term NMP. This method is particularly beneficial for low-flow organs and preserves the natural interdependence of organ systems. By recovering and perfusing multiple organs together within a single circuit, this approach supports their interactions, potentially enabling extended ex situ storage. It also facilitates sequential organ harvesting for transplantation and offers a valuable platform for drug discovery and testing. Additionally, it may significantly benefit multivisceral transplant recipients, among the most complex cases in transplantation.

To position ex situ multivisceral NMP within the broader landscape of healthcare innovation, it is essential to consider recent advancements in modular and connected health technologies [13]. The integration of Internet of Things (IoT) platforms into the remote management of complex patients—particularly those with chronic neurological or metabolic conditions—illustrates the growing emphasis on real-time monitoring, data integration, and adaptive system design [14]. These developments closely parallel the requirements of ex situ organ support, where continuous, multi-parameter data acquisition and system responsiveness are vital for maintaining physiological stability. Such technologies have demonstrated clear clinical value in high-dependency, dynamic care settings and offer a conceptual framework for enhancing the performance of multivisceral perfusion systems. Applying these principles to long-term, multi-organ perfusion could improve system efficiency, reduce costs compared to isolated single-organ platforms, and enable broader translational applications.

Research on multivisceral perfusion is still limited [15,16,17,18]. Therefore, the goal in this proof-of-concept study was to develop a multivisceral perfusion platform using a rapid-prototyping and iterative design approach. The primary objective of this study was to create a stable platform for multivisceral organ perfusion. The secondary objectives were to characterize perfusion dynamics, physiological parameters, and basic molecular and histological data from this iterative design process. Initial success was deemed to be the provision of steady state perfusion for at least eight hours.

## 2. Materials and Methods

### 2.1. Rationale for Iterative Design Process

An iterative design approach was adopted for developing the multivisceral perfusion platform, allowing continuous refinement through cycles of design, testing, and review. Significant modifications were made with each cycle, enabling early identification of issues and reducing the number of animals used. This method allowed for targeted improvements based on real-time experimental findings, enhancing the platform’s functionality and reliability.

### 2.2. Generation 1 Perfusion Set-Up (Gen 1)

**Figure 1:**
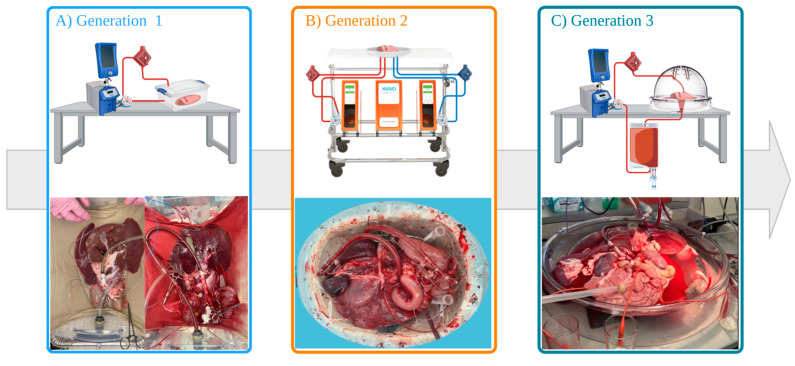
Global overview of design process and each generation of the setups used.

The first-generation platform featured a custom-built open perfusion circuit designed to maintain continuous flow and oxygenation to the organs. The circuit consisted of a centrifugal pump (Bio-Console 560, Medtronic, Minneapolis, MN, USA), a flow sensor, an oxygenator (LivaNova, London, UK) connected to a water bath (Gentherm, Novi, MI, USA), and a disposable TruWave pressure transducer (Edwards Lifesciences, Irvine, CA, USA) linked to the aortic cannula (Figure 1A). Organs were placed in a plastic basin (Walmart, Bentonville, AR, USA) and stabilized with saline-soaked towels to prevent twisting during perfusion.

The multi-visceral organ blocks (*n* = 5), containing the liver, kidneys, pancreas, spleen, and a segment of the duodenum, were obtained from Yorkshire pigs at a local abattoir (following the U.S. Animal Welfare Policy), simulating a donation after circulatory death (DCD) model. Following exsanguination, blood was collected into heparinized canisters (25,000 IU). A thoraco-laparotomy was performed, and the organs were cold flushed (Table A1). The abdominal and thoracic cavities were packed with ice for topical cooling. The flush was continued until it returned clear, signaling the cessation of perfusion. The abdominal organs were removed *en bloc*, and the length of the small bowel was reduced to a 20–30 cm duodenal segment. The supra-celiac aorta was cannulated with a 28 Fr cannula, and the bile duct and ureters were cannulated with 8 Fr cannulas to drain bile and urine.

HMP was initiated to clear microthrombi and recharge ATP levels prior to NMP [19]. After two hours of HMP with a glucose-enriched cold storage solution, the organs were transitioned to NMP. The perfusate for NMP consisted of autologous blood and supplements (Table A1). An infusion of sodium taurocholate (0.187 g/mL), heparin (833.33 U/mL), and Veletri (8.33 µg/mL) were all administered at 1 mL/h. Oxygenation was maintained with 100% oxygen at a flow rate of 500 mL/min.

This set-up yielded suboptimal perfusion parameters including significant perfusate loss, bowel edema, and low urine production during perfusion.

### 2.3. Generation 2 Perfusion Set-Up (Gen 2)

In response to the limitations observed in generation 1, the second iteration of the platform implemented several key changes. A dual perfusion system was introduced, using separate cannulas and pumps for the hepatic artery and portal vein (*n* = 3). The Liver Assist device (XVIVO B.V., Groningen, The Netherlands) was repurposed for this setup, allowing for more controlled perfusion of the portal and arterial circuits (Figure 1B). Pulsatile perfusion for the aorta was introduced.

To reduce warm ischemia time and improve the quality of blood used during perfusion, organs for pig 8 and onwards were retrieved from anesthetized in-house Yorkshire pigs rather than abattoir-sourced pigs. The surgical protocol is extensively described in Appendix A. During procurement, a thoraco-laparotomy was performed, and 30,000 units of heparin was administered to ensure complete heparinization before blood collection. The infra-renal aorta was encircled, and the supra-hepatic cava was prepared for cannulation. The blood was collected in a controlled manner by passive drainage into a sterile collection bag pre-lined with 25,000 units of heparin, aided by tilt augmentation of the pig. After confirming death, a flush was initiated (Table A1). Back table preparation was similar to Gen 1, apart from portal vein cannulation with a 24 Fr cannula.

NMP commenced after back table prep, omitting the HMP step to keep the focus on optimizing the NMP process. Dual perfusion was maintained throughout, with a separate pump controlling flow to the portal vein and aortic circuits. The perfusate composition was similar to Gen 1 (Table A1).

The main limitation with this set-up was the maximum arterial flow of 1 L/min afforded by the Liver Assist device. Bowel edema and fluid loss persisted, although to a lesser degree than in Gen 1.

### 2.4. Generation 3 Perfusion Set-Up (Gen 3)

In generation 3, we reverted to single continuous perfusion through the supra-celiac aorta (*n* = 4) (Figure 1C). The Liver Assist device was replaced with a custom-designed circuit, similar to Gen 1. The major changes were the addition of a separate reservoir (LivaNova, London, UK) and a repurposed organ dome (XVIVO, Gothenburg, Sweden) that allowed for better organ arrangement.

Organ blocks for Gen 3 were similarly recovered from in-house pigs as described for Gen 2. The aorta was prepared for cannulation via the thoracic aorta, and the small bowel was preserved up to 2.25 m in length. NMP was maintained for 8–12 h. The perfusate composition remained consistent with previous generations (Table A1).

### 2.5. Logging of Perfusion Parameters and Viability Assessment

Perfusate sampling was done via the arterial sample port and graft IVC and taken at regular intervals during perfusion, while biopsies from each organ were taken pre- and post-perfusion. Flow rate, mean arterial pressures (MAP), resistance, perfusate temperature, and urine output were continuously monitored and recorded. Electrolytes and blood gas values were measured via blood gas analysis using an iSTAT (ABBOTT, Chicago, IL, USA). The oxygen consumption was calculated as followed:

$$\begin{array}{c} \mathrm{Oxygen}\;\mathrm{consumption}\;({\mathrm{mlO}}_{2}/\min^{-1})\\ =(((Hb\times 2.4794)+(pO2arterial\times K))-((0.024794\times Hb\times SO2venous)+(pO2venous\times K)))\times Q \end{array}$$

Hb: hemoglobin content (mmol/L).
pO_2_: partial oxygen pressure (kPa).
K: solubility constant of oxygen in H_2_O at 37 °C (0.0225 mlO_2_ per kPa).
SO_2_: hemoglobin saturation (%).
Q: blood flow (L/min).

### 2.6. Clinical Chemistry

Clinical chemistry analyses for alanine aminotransferase (ALT), aspartate aminotransferase (AST), alkaline phosphatase (ALP), lactate dehydrogenase (LDH), creatinine, blood urea nitrogen (BUN), potassium, lipase, and amylase were conducted routinely.

### 2.7. Histological Analysis

Biopsies were obtained from the renal cortex, liver parenchyma, pancreas, and the luminal wall of the duodenum, and fixed in 4% formalin. These samples were embedded in paraffin wax, sectioned at 4 µm, and stained with hematoxylin and eosin (H&E) to highlight morphological features. Histological preparation was performed, and sections were evaluated and scored in a blinded manner by a board-certified pathologist using established scoring protocols [20,21,22,23,24].

### 2.8. Data and Statistical Analysis

GraphPad Prism (version 9.1.0) was used to visualize the data and perform statistical analysis. Longitudinal perfusion data per generation are shown as single values over time in the figures and the overall arithmetic mean with the standard deviation (SD). Differences across experimental groups were analyzed using one-way analysis of variance (ANOVA) followed by Tukey’s multiple comparisons. Injury markers are expressed as aligned scatter plots and the arithmetic mean with the SD. Differences across experimental groups were assessed using a two-way ANOVA with Fisher’s LSD test. All statistical tests were two-tailed, and differences between groups were considered statistically significant when *p* < 0.05. No experimental units or data points were excluded from the analyses.

## 3. Results

### 3.1. Preservation Characteristics

The preservation parameters for each pig are summarized in Table 1. Gen 3 achieved a significant reduction in warm ischemia time (WIT) compared to Gens 1 and 2. Cold ischemia time was notably longer in Gen 1 than in Gens 2 and 3. HMP was performed exclusively in Gen 1. No significant differences were observed in the durations of NMP across the different generations. However, MAP during NMP in Gen 1 was significantly higher than that in the other generations.

### 3.2. Generation 1 Perfusion

**Figure 2:**
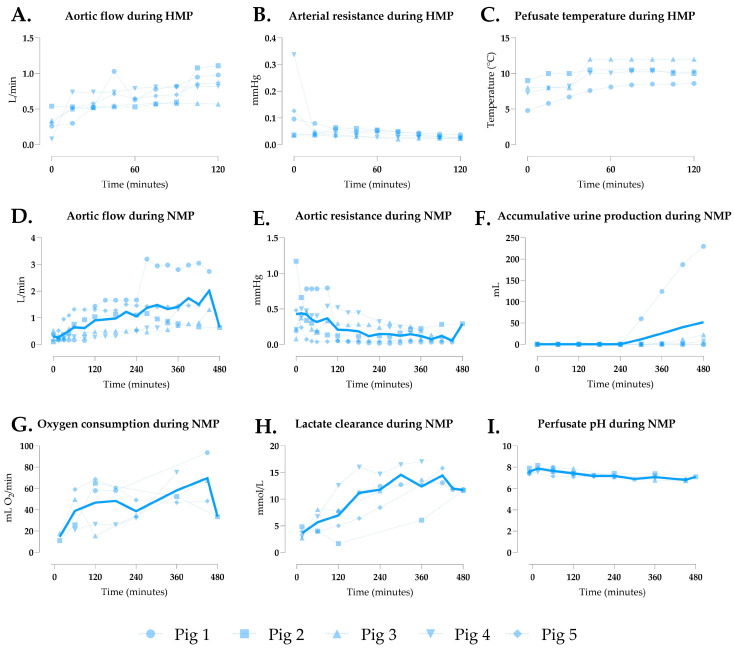
Perfusion parameters of multivisceral grafts in Gen 1. (**A**) Aortic flow, (**B**) aortic resistance, and (**C**) graft temperature during hypothermic machine perfusion (HMP). (**D**) Aortic flow, (**E**) aortic resistance, (**F**) accumulative urine production, (**G**) oxygen consumption, (**H**) lactate clearance, and (**I**) perfusate pH during normothermic machine perfusion (NMP). Data are shown as individual values (dotted lines) and mean (continuous line).

In the initial setup, oxygenated HMP preceded NMP, with HMP parameters shown in Figure 2A–C. Aortic resistance decreased steadily, reaching 0.028 ± 0.006 after 120 min (Figure 2B). The NMP hemodynamics are detailed in Figure 2D–E, with grafts showing physiological flow rates (mean 1.018 ± 0.506 L/min, Figure 2D) and generally declining resistance, except in pigs 1 and 5 (Figure 2E). Urine output was low overall, averaging 51.90 ± 99.97 mL (Figure 2F), except in pig 1. Oxygen consumption fluctuated (Figure 2G), while rising lactate levels (Figure 2H) and declining pH (Figure 2I) indicated suboptimal perfusion.

### 3.3. Generation 2 Perfusion

**Figure 3:**
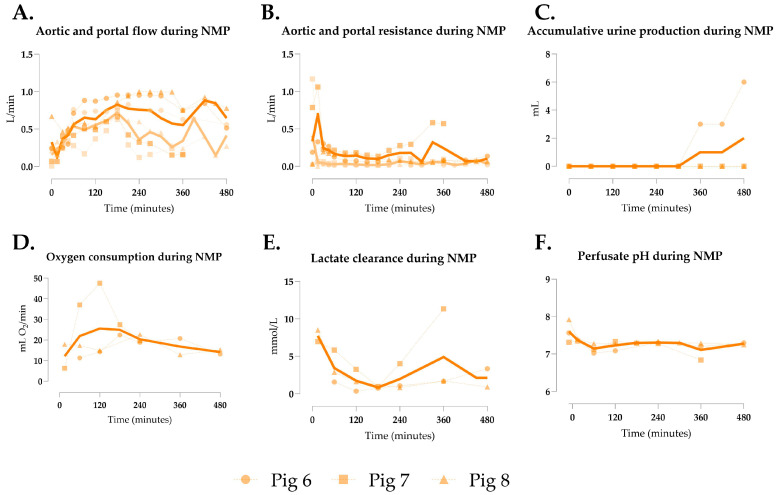
Perfusion parameters of multivisceral grafts in Gen 2. (**A**) • Aortic flow and • portal flow, (**B**) • aortic and • portal resistance, (**C**) accumulative urine production, (**D**) oxygen consumption, (**E**) lactate clearance, and (**F**) perfusate pH during normothermic machine perfusion (NMP). Data shown as individual values (dotted lines) and mean (continuous line).

Rising lactate levels in the first generation suggested inadequate perfusion, likely due to reduced mesenteric venous return from bowel shortening, causing portal hypo-perfusion. To address this, NMP with dual arterial and portal perfusion was implemented. Mean aortic flow was 0.617 ± 0.201 L/min, limited to 1 L/min by machine constraints, yielding in significant lower flow rates than Gen 1 (Figure 3A/Table 2). Portal flow averaged 0.427 ± 0.228 L/min (Figure 3A), with aortic and portal resistances of 0.195 ± 0.148 and 0.062 ± 0.170, respectively (Figure 3B). Only pig 6 produced urine (Figure 3C). Oxygen consumption was significantly lower than in Gen 1 (Figure 3D/Table 2). Lactate clearance was achieved in two pigs, with significantly lower mean levels than in Gen 1 (Figure 3E/Table 2), and perfusate pH remained stable (Figure 3F). Bowel peristalsis was observed in all shortened segments (Supplementary Video S1).

### 3.4. Generation 3 Perfusion

**Figure 4:**
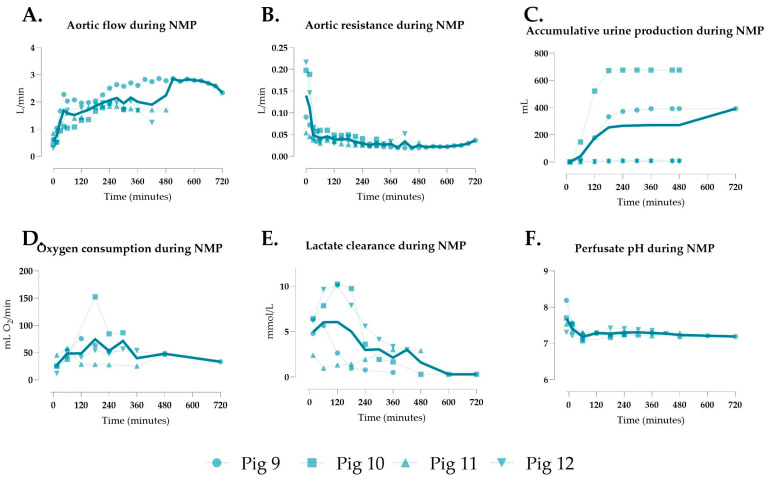
Perfusion parameters of multivisceral grafts using Gen 3. (**A**) Aortic flow, (**B**) aortic resistance, (**C**) accumulative urine production, (**D**) oxygen consumption, (**E**) lactate clearance, and (**F**) perfusate pH during normothermic machine perfusion (NMP). Data are shown as individual values.

In the third generation, single continuous perfusion was used with a longer intact small bowel segment (2.25 m) to minimize portal hypoperfusion. Mean aortic flow was 2.089 ± 0.633 L/min, significantly higher than in Gens 1 and 2 (Figure 4A/Table 2), with aortic resistance markedly lower at 0.038 ± 0.027 (Figure 4B/Table 2). Urine production occurred in all pigs (Figure 4C). Oxygen consumption fluctuated but was significantly higher than in Gen 2 (Figure 4D/Table 2). Lactate clearance was achieved in four of five pigs, with significant lower mean levels than in Gen 1 (Figure 4E/Table 2), and perfusate pH remained stable (Figure 4F). Bowel peristalsis was observed during all perfusions (Supplementary Video S2).

### 3.5. Cellular Injury During Multivisceral Perfusion

**Figure 5:**
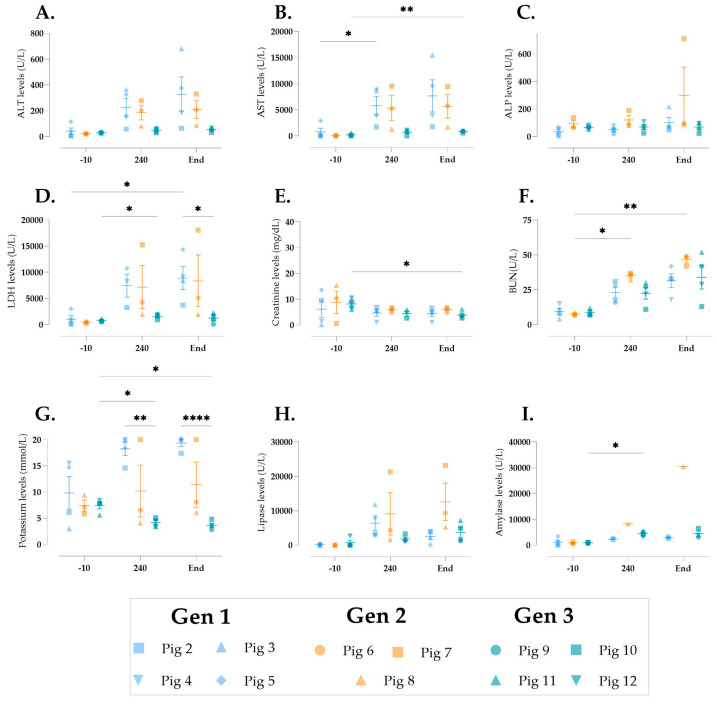
Cellular injury during multivisceral perfusion. (**A**) alanine aminotransferase (ALT), (**B**) aspartate aminotransferase (AST), (**C**) alkaline phosphatase (ALP), (**D**) lactate dehydrogenase (LDH), (**E**) creatinine, (**F**) blood urea nitrogen (BUN), (**G**) potassium, (**H**) lipase, and (**I**) amylase were measured at baseline (−10), 4 h into perfusion (240), and at the end of perfusion (end). Data are expressed as aligned scatter plots and the arithmetic mean + SD. * *p* < 0.05, ** *p* < 0.01, **** *p* < 0.0001.

To assess cellular injury during multivisceral perfusion, we analyzed biomarkers of organ function. Liver enzymes ALT, AST, and ALP were measured (Figure 5A–C). AST increased significantly in Gens 1 and 3 (Figure 5B), though Gen 3 pigs showed the lowest ALT and AST levels at the end of perfusion. ALP remained stable, except for an increase in pig 7 (Figure 5C). LDH, a marker of tissue damage, rose significantly in Gens 1 and 3 but was lower at the end of perfusion in Gen 3 compared to Gen 1 (Figure 5D). Renal function was assessed by creatinine clearance and BUN. Perfusate creatinine levels decreased significantly in Gen 3 (Figure 5E), while BUN increased significantly over time (Figure 5F). Potassium levels decreased significantly in Gen 3 and were lower than in Gen 1 (Figure 5G). Pancreatic markers lipase and amylase were also measured (Figure 5H,I). Amylase was higher after 240 min in Gen 3 but not at the end of perfusion.

### 3.6. Histological Changes over Time

**Figure 6:**
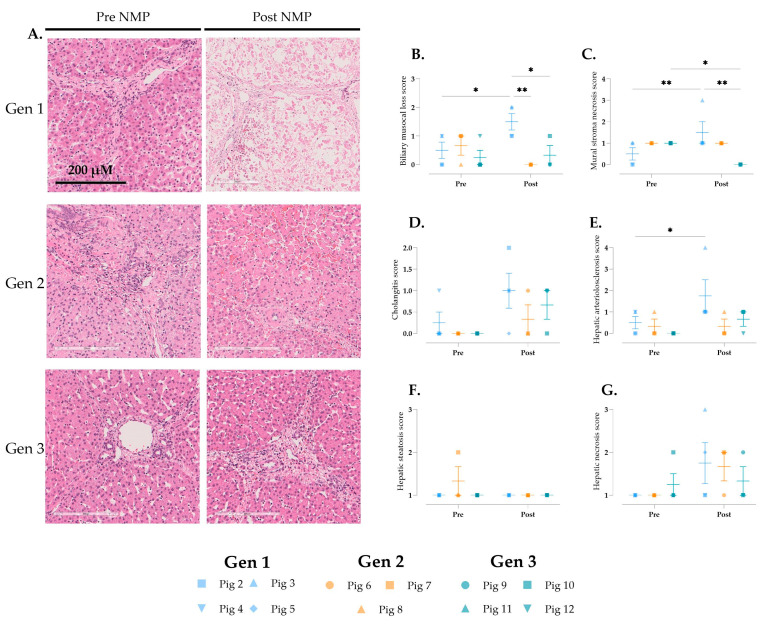
Histological assessment of the liver during multi-visceral perfusion. (**A**) Liver parenchyma hematoxylin and eosin-stained sections representing pre- and post-NMP (Gen 1 = pig 3, Gen 2 = pig 8, and Gen 3 = pig 12). (**B**) biliary mucosal loss, (**C**) mural stromal necrosis, (**D**) cholangitis, (**E**) hepatic arteriosclerosis, (**F**) hepatic steatosis, and (**G**) hepatic necrosis were scored. Data are expressed as aligned scatter plots and the arithmetic mean + SD. * *p* < 0.05, ** *p* < 0.01. Original magnification: 20×, scale bar is 200 µM.

**Figure 7:**
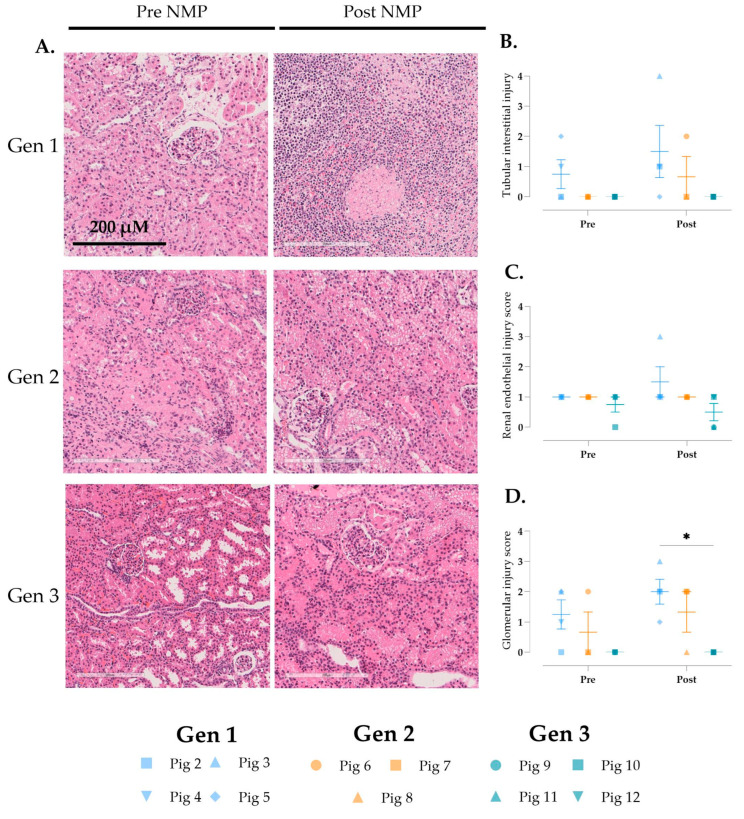
Histological assessment of the kidney during multi-visceral perfusion using the EGTI scoring system. (**A**) Renal cortex hematoxylin and eosin-stained sections representing pre- and post-NMP (Gen 1 = pig 3, Gen 2 = pig 8, and Gen 3 = pig 12). (**B**) Glomerular injury score, (**C**) renal endothelial injury, and (**D**) tubular interstitial were scored. Data are expressed as aligned scatter plots and the arithmetic mean + SD. Original magnification: 20×, scale bar is 200 µM. * *p* < 0.05.

**Figure 8:**
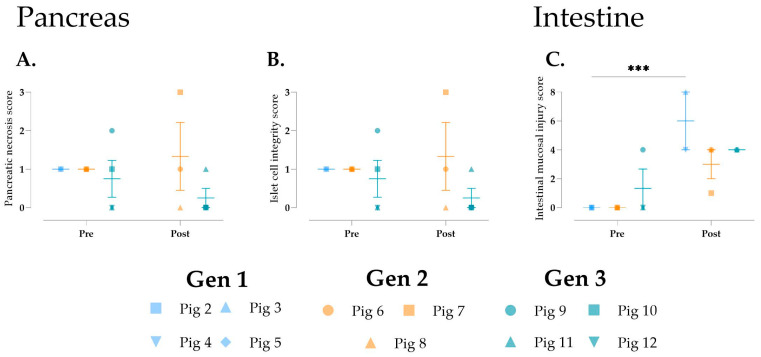
Histological assessment of the pancreas and intestine during multi-visceral perfusion. (**A**) Pancreatic necrosis score, (**B**) islet cell integrity, and (**C**) intestinal musical injury were scored pre- and post-NMP. Data are expressed as aligned scatter plots and the arithmetic mean + SD. *** *p* < 0.001.

Histological changes in the liver, kidneys, pancreas, and intestine were analyzed both before and after perfusion (Figure 6, Figure 7 and Figure 8). Notable differences in liver tissue were primarily observed in Gen 1, in which histological scoring using the Modified Hansen Score [20] revealed significantly greater biliary mucosal loss, hepatic arteriosclerosis, and mural stroma necrosis following perfusion (Figure 6B,C,E). Additionally, Gen 1 resulted in increased cholangitis and hepatic necrosis, although these increases were not statistically significant (Figure 6D,G). In Gens 2 and 3, non-significant increases in biliary inflammation and hepatic necrosis were also noted (Figure 6D,G).

Histological evaluation of the kidneys using the EGTI scoring system [23] displayed more inflammation in Gen 1 and minimal edema in all groups post-perfusion (Figure 7A). No differences were observed when looking at tubulo-interstitial and endothelial injury (Figure 7B,C). A significantly higher glomerular injury score in Gen 1 compared to Gen 3 was observed (Figure 7D).

No significant changes were observed in the pancreas, although fat/parenchymal necrosis and islet cell integrity appeared to improve following perfusion in Gen 3 (Figure 8A,B). Lastly, intestinal mucosal injury, as assessed by the Chiu/Park score [24], significantly worsened after perfusion in Gen 1 (Figure 8C).

## 4. Discussion

### 4.1. Study Strengths and Key Findings

The current organ shortage highlights the urgent need for advancements in machine preservation techniques, particularly in normothermic perfusion of the kidney, pancreas, and small bowel. This study aimed to develop a multivisceral perfusion platform that systematically characterizes perfusion dynamics, physiological parameters, and molecular and histological outcomes through an iterative design process. We demonstrated the feasibility of abdominal multivisceral NMP for up to 8 h, achieving stable arterial flow, perfusate pH, and sufficient oxygen consumption, all indicating organ viability.

A key strength of our study lies in the iterative design approach, which allowed for continuous, data-driven refinements. Unlike a traditional linear design process, in which designs are finalized before testing, our approach enabled real-time problem solving. For instance, after identifying excessive bowel edema and fluid loss in Gen 1, we introduced significant changes in Gen 2, including shortening the small bowel and implementing dual-circuit perfusion for the hepatic and portal systems. These adjustments improved portal flow, and enhanced lactate clearance. Gen 3 further improved outcomes by adopting single continuous perfusion with higher flow rates and a redesigned basin for optimal organ arrangement. This process not only yielded better results but also reduced the number of animals required by addressing issues early on.

The iterative design of our system enabled optimization of critical parameters such as flow rates, pressures, and organ stability, advancing the standardization of multivisceral perfusion protocols. While optimal parameters for abdominal NMP remain undefined, we identified conditions supporting multi-organ viability. In our third-generation system, arterial flow rates of 0.8–1.7 L/min facilitated effective lactate clearance and stable perfusate pH. Single arterial perfusion outperformed dual arterial and portal perfusion, potentially due to the 1 L/min arterial flow limitation in the latter. A MAP of 60–75 mmHg supported tissue oxygenation, urine production, and biochemical stability, though individual organs, such as the pancreas or small intestine, may benefit from tailored arterial pressures. Further investigation is needed to refine these parameters.

While urine production improved in Gen 3, the composition of urine and its relation to kidney histology were not fully evaluated. We did observe a decrease in perfusate creatinine levels, while BUN levels only increased slightly, reflecting proper kidney function. Furthermore, the post-NMP kidney histology and macroscopic appearance remained similar to pre-NMP.

Amylase and lipase levels rose slightly, with no histological signs of pancreatic injury post-NMP, suggesting minimal pancreatic damage. These levels were considerably lower than those reported in isolated porcine pancreas perfusion [25,26,27]. Currently, NMP of pancreas grafts has been minimally explored, with perfusion times often restricted by graft edema and injury [10,22]. Pancreas perfusion remains underexplored, but our results indicate that multivisceral abdominal perfusion may offer an effective approach for preserving the pancreas ex situ.

Intestinal peristalsis, a key indicator of gut function, was consistently observed in Gen 3 [28]. While intrinsic to the gastrointestinal tract, its occurrence during normothermic perfusion is rarely reported [12,29]. Histological analysis revealed increased intestinal injury scores post-NMP, although previous studies on isolated porcine intestinal perfusion showed lower injury scores with shorter (6 h) perfusions at sub-normothermic temperatures [11,12]. Significant bowel edema and fluid loss in our study limited perfusion to 7–12 h, likely due to ischemia-reperfusion injury (IRI) compromising luminal integrity. Intestinal grafts from DCD donors are especially vulnerable to IRI, rendering them generally unsuitable for transplantation [30]. Since all grafts in this study were exposed to WIT, this likely contributed to the intestinal injury. Potential improvements for intestinal preservation include a cold luminal flush, extended bowel length, better flow and pressure control, and nutrient supplementation during perfusion.

### 4.2. Preclinical Evidence in Multivisceral Organ Perfusion Research

The feasibility of multivisceral perfusion was first demonstrated in studies by Prieto et al. (1988) [15] and Chien et al. (1989) [16], in which stable perfusion of multiple organs (heart, lungs, liver, kidneys, pancreas) was achieved for up to 22 and 37 h, respectively. More recent studies by Chen et al. (2021) [17] and Stevens et al. (2024) [18] have explored perfusing mini pig and butcher pig abdominal organs for 7–10 h using single and dual-circuit perfusion. However, these studies involved significant variations in perfusion protocols, with the early studies focusing on heart and lung perfusion at lower temperatures and minimal intestinal involvement. By contrast, Chien did not subject grafts to cold ischemia, while Stevens worked with butcher pigs, during which significant warm ischemia is inevitable. Moreover, none of these studies reported detailed histological assessments, which are critical for evaluating tissue integrity. Both Chien and Stevens perfused longer bowel segments and observed bowel edema along with intestinal fluid loss. Moreover, none of the groups demonstrated lactate clearance, and all reported elevated ALT and AST levels, indicating suboptimal liver viability during multivisceral perfusion.

### 4.3. Experimental Considerations, Study Limitations, and Future Directions

Pigs were selected as the animal model because of their anatomical and physiological similarities to humans [31]. This makes them particularly suitable for studies involving machine perfusion and surgical techniques, as clinical-grade devices and instruments can be readily applied. While perfusion studies have shown that porcine models are highly translatable [32], they do not replicate the chronic injury commonly observed in human donor organs. Ultimately, these experiments should be repeated using donor multivisceral grafts that are deemed unsuitable for transplantation.

While this study provides valuable insights, several limitations should be acknowledged, particularly regarding variability across experimental generations. Early experiments using abattoir-sourced pigs introduced variability in ischemia times and perfusate quality. The decision to use abattoir pigs aimed to minimize animal suffering during the initial exploratory phase. In the first generation of experiments, HMP was incorporated due to its well-documented clinical benefits [19,33]. However, the decision to omit HMP in subsequent experiments was made to focus on refining the NMP process. Slight differences in perfusate composition were due to each graft its individual needs. Additionally, the small sample size reflects the significant resources required for these experiments, and the limited blood volume restricted perfusion duration due to fluid losses. Team expertise also evolved over the course of the study. These differences make it challenging to pinpoint which specific changes yielded the greatest benefits, complicating direct comparisons between generations. Nonetheless, the findings underscore the feasibility of multivisceral perfusion and provide a solid foundation for further research and optimization.

This study served as a proof of concept. Future investigations should incorporate the refined experimental setup, a larger sample size, immunological assessment, and ultimately, transplantation of the perfused organs. However, this presents a significant challenge, as an autotransplant model is not feasible, and for assessment of each individual organ, each organ would need to be transplanted into a separate recipient pig. Additionally, extending the duration of perfusion should be explored to achieve clinical translation, by increasing the circulating blood volume and minimizing fluid losses. This could be achieved by modifying the blood collection technique to improve yield, using a pig donor or previously banked autologous blood, or selecting a larger pig. Further optimization of the perfusate’s colloid osmotic pressure (COP) is also crucial to reduce edema and maintain fluid balance across the graft. In addition, maintaining vascular tone using vasopressors and a pulsatile pump should be explored. Lastly, comparisons to single-organ NMP models should be considered.

### 4.4. Clinical Perspectives of Multivisceral Perfusion

The success of in situ multi-organ perfusion with NRP has demonstrated significant benefits for organ resuscitation and post-transplant outcomes [34]. However, the duration of NRP is constrained by ethical and logistical limitations, highlighting a critical opportunity for ex situ multivisceral perfusion. This emerging approach holds great promise in transplantation medicine, offering the ability to preserve, assess, and treat multiple abdominal organs simultaneously outside the body. By enabling continuous real-time physiological and metabolic monitoring, it allows for more accurate evaluation of organ viability, prolonged preservation times, and the delivery of targeted therapeutic interventions. The integration of IoT technologies into perfusion platforms could further enhance monitoring and control by enabling remote data acquisition, automation, and early detection of graft deterioration. Clinically, multivisceral perfusion could revolutionize the management of marginal or extended criteria donor organs, increase the utilization of available grafts, and improve outcomes in complex multi-organ transplants. As the field evolves, successful translation into clinical practice will require multidisciplinary collaboration, standardized protocols, and strong evidence of safety and efficacy.

## 5. Conclusions

This technique shows great promise for enhancing organ preservation and enabling resuscitation before transplantation. Extending perfusion durations to 2–3 weeks could create a paradigm of “organs on demand,” mitigating the time pressures of organ allocation. Organs could be separated from the bloc, individually preserved, and transported to recipient centers when ready for transplantation. Ultimately, multivisceral perfusion has the potential to bridge the supply–demand gap in transplantation and significantly improve patient outcomes.

## Figures and Tables

**Table 1:** Preservation characteristics for each group shown as the mean + SD.

Demographics	Experimental Groups			*p*-Value		
	Gen 1	Gen 2	Gen 3	G1 vs. G2	G1 vs. G3	G2 vs. G3
WIT (min)	9.4 ± 3.1	8.3 ± 1.2	3.3 ± 1.7	0.8086	0.0085	0.0447
Cold flush (min)	11.8 ± 0.8	18.7 ± 13.9	17.3 ± 2.5	0.3812	0.4774	0.9590
CIT (min)	368.6 ± 55.8	265.3 ± 61.9	184.8 ± 32.3	0.0506	0.0011	0.1497
Duration of HMP (min)	110.8 ± 21.78	-	-	-	-	-
Duration of NMP (min)	438.0 ± 47.6	440.0 ± 69.3	480.0 ± 176.6	0.9997	0.8438	0.8873
Mean MAP during NMP (mmHg)	110.3 ± 22.31	73.15 ± 13.32	63.60 ± 7.565	<0.0001	<0.0001	0.0956

WIT: warm ischemia time, CIT: cold ischemia time, HMP: hypothermic machine perfusion, NMP: normothermic machine perfusion, MAP: mean arterial pressure.

**Table 2:** Mean aortic flow, resistance, total urine output, oxygen consumption, lactate clearance, and perfusate pH of grafts in the different experimental groups over time. Shown as mean + SD.

NMP Parameters	Experimental Groups			*p*-Value		
	Gen 1	Gen 2	Gen 3	G1 vs. G2	G1 vs. G3	G2 vs. G3
Aortic flow (L/min)	1.018 ± 0.506	0.617 ± 0.201	2.089 ± 0.633	0.0498	<0.0001	<0.0001
Aortic resistance	0.224 ± 0.127	0.195 ± 0.148	0.038 ± 0.027	0.6916	<0.0001	<0.0001
Total urine output (mL)	51.90 ± 99.97	2 ± 3.46	271.3 ± 324.8	0.9375	0.278	0.233
Oxygen consumption (mL O_2_/min)	43.56 ± 16.57	19.42 ± 5.21	49.52 ± 15.79	0.0082	0.6583	0.0009
Lactate clearance (mmol/L)	10.44 ± 3.72	3.26 ± 2.37	3.10 ± 2.21	<0.0001	<0.0001	0.993
Perfusate pH	7.27 ± 0.34	7.29 ± 0.14	7.30 ± 0.13	0.9843	0.961	0.9961

## Data Availability

Data available upon request.

## Supplementary Materials

The following supporting information can be downloaded at: https://www.mdpi.com/article/10.3390/jcm14134620/s1, Video S1: Peristalsis observed during Gen 2 perfusions; Video S2: Peristalsis observed during Gen 3 perfusions.

## Abbreviations

The following abbreviations are used in this manuscript:

ALP	Alkaline phosphatase
ALT	Alanine aminotransferase
ANOVA	Analysis of variance
AST	Aspartate aminotransferase
BUN	Blood urea nitrogen
DCD	Donation after circulatory death
H&E	Hematoxylin and eosin
HMP	Hypothermic machine perfusion
IRI	Ischemia-reperfusion injury
IVC	Inferior vena cava
LDH	Lactate dehydrogenase
MAP	Mean arterial pressure
NMP	Normothermic machine perfusion
NRP	Normothermic regional perfusion
WIT	Warm ischemia time

## Appendix A

### Surgical Protocol at the In-House Animal Facility

The preparation and surgical procedures for the animals prior to the experimental procedures were similar for all pigs. They were housed in pens with enrichment available. The animals were fasted for 24 h prior to surgery. The animals were sedated with tiletamine-zolazepam at a dosage of 5–6 mg/kg. An intravenous (IV) catheter was then placed via the ear vein, and vital signs, including heart rate, SpO_2_, and temperature, were collected. Based on the animal’s size and weight, an appropriately sized endotracheal tube was selected. The animal was then intubated in the supine position, and the tube was secured to ensure a patent airway. After intubation, the animal was transferred to the operating room (OR) space, and connected to sensors to continuously monitor heart rate, SpO_2_, and respiration. Finally, the animal was placed on ventilation with isoflurane at a concentration of 1–3% and oxygen at 2.5 L/min. All animals were euthanized while under general anesthesia.

**Table A1:** Pig and graft characteristics and perfusion composition for each experiment. NR: not recorded.

	Pig 1	Pig 2	Pig 3	Pig 4	Pig 5	Pig 6	Pig 7	Pig 8	Pig 9	Pig 10	Pig 11	Pig 12
**Generation set-up**	1	1	1	1	1	2	2	2	3	3	3	3
**Pig type**	Butcher	Butcher	Butcher	Butcher	Butcher	Butcher	Butcher	Laboratory	Laboratory	Laboratory	Laboratory	Laboratory
**Pig age (months)**	6	6	8	6	6	6	5	3	3.5	3	3	3
**Pig sex**	Female	Female	Female	Male	Female	Male	Male	Male	Male	Male	Male	Male
**Pig weight (Kg)**	NR	NR	NR	NR	NR	NR	NR	46.4	50.9	33.8	37	39.9
**Flush type**	3L NaCl + 4L HTK	3L NaCl + 4L Servator B™ Safe	3L NaCl + 4L HTK	3L NaCl + 4L HTK	3L NaCl + 4L HTK	6L NaCl	3L NaCl + 4L HTK	3L NaCl + 4L HTK	3L NaCl + 4L HTK	3L NaCl + 4L HTK	6L HTK	6L HTK
**CIT (hours)**	5:55	5:45	5:57	5:21	7:45	4:58	5:04	3:14	2:52	3:29	3:33	2:25
**Bowel length (m)**	0.03	0.03	0.02	0.1–0.3	0.1–0.3	0.03	0.03	0.03	2.25	2.25	2.25	2.25
**Graft weight pre NMP (g)**	NR	1013	1453	NR	NR	NR	NR	1900	1800	2550	1800	2000
**Perfusate composition NMP**												
**Whole blood (mL)**	1000	1000	1000	1600	1900	1250	1300	1800	1775	2100	1550	1670
**Cefuroxim in 20 mL NaCl (g)**	1.5	1.5	1.5	1.5	1.5	1.5	1.5	1.5	1.5	1.5	1.5	1.5
**Calcium gluconate (mL)**	10	20	20	20	10	20	30	20	20	20	20	20
**TPN (mL)**	30	30	30	30	70	30	36	38	30	30	30	30
**Albumin 5% (mL)**	250	250	250	250	1000	700	750	250	250	250	750	500
**Albumin 25% (mL)**	-	-	-	-	-	-	-	-	200	-	100	100
**Creatinine (mg)**	218	218	218	218	218	218	218	218	218	218	218	218
**Mannitol (g)**	5	5	5	2.5	4	4	4	2.5	2.5	2.5	0.1	25
**Sodium Nitroprusside (mg)**	5	17.5	10	20	22	-	-	-	-	-	-	-
**Velitri (ug)**	83.3	-	-	-	-	164.93	-	83.3	83.3	49.98	-	16.66
**8.4% NaHCO₃ (mL)**	30	30	40	60	85	70	30	-	20	15	15	35
**Fluid loss replacement (5% albumin) (mL)**	-	320	100	700	-	450	-	250	2750	1900	250	2510
